# Implementing knowledge into practice for improved neonatal survival; a cluster-randomised, community-based trial in Quang Ninh province, Vietnam

**DOI:** 10.1186/1472-6963-11-239

**Published:** 2011-09-27

**Authors:** Lars Wallin, Mats Målqvist, Nguyen T Nga, Leif Eriksson, Lars-Åke Persson, Dinh P Hoa, Tran Q Huy, Duong M Duc, Uwe Ewald

**Affiliations:** 1 Department of Neurobiology, Care Sciences and Society, Division of Nursing, Karolinska Institutet, SE-171 76 Stockholm, Sweden; 2 International Maternal and Child Health (IMCH), Department of Women's and Children's Health, Uppsala University, SE-751 85 Uppsala, Sweden; 3 Vietnam Sweden Uong Bi General Hospital, Quang Ninh, Việt Nam; 4 Hanoi School of Public Health, 138 Giảng Võ St., Ba ĐÌnh District, Hà Nội, Việt Nam; 5 Ministry of Health, 138A Giảng Võ St., Ba ĐÌnh District, Hà Nội, Việt Nam; 6 Neonatology, Department of Women's and Children's Health, Uppsala University, SE-751 85 Uppsala, Sweden

## Abstract

**Background:**

Globally, almost 4 million newborns die during the first 4 weeks of life every year. By increased use of evidence-based knowledge in the healthcare system a large proportion of these neonatal deaths could be prevented. But there is a severe lack of knowledge on effective methods for successful implementation of evidence into practice, particularly in low- and middle-income countries. Recent studies have demonstrated promising results with increased survival among both mothers and newborns using community-based approaches. In Vietnam evidence-based guidelines on reproductive health were launched in 2003 and revised in 2009. The overall objective of the current project is to evaluate if a facilitation intervention on the community level, with a problem-solving approach involving local representatives if the healthcare system and the community, results in improvements of neonatal health and survival.

**Methods/Design:**

The study, which has been given the acronym NeoKIP (Neonatal Health - Knowledge Into Practice), took place in 8 districts composed by 90 communes in a province in northern Vietnam, where neonatal mortality rate was 24/1000 in 2005. A cluster randomised design was used, allocating clusters, as defined as a commune and its correponding Commune Health Center (CHC) to either intervention or control arm. The facilitation intervention targeted staff at healthcare centres and key persons in the communes. The facilitator role was performed by lay women (Women's Union representatives) using quality improvement techniques to initiate and sustain improvement processes targeting identified problem areas. The intervention has been running over 3 years and data were collected on the facilitation process, healthcare staff knowledge in neonatal care and their behaviour in clinical practice, and reproductive and perinatal health indicators. Primary outcome is neonatal mortality.

**Discussion:**

The intervention is participatory and dynamic, focused on developing a learning process and a problem-solving cycle. The study recognises the vital role of the local community as actors in improving their own and their newborns' health, and applies a bottom-up approach where change will be accomplished by an increasing awareness at and demand from grass root level. By utilising the existing healthcare structure this intervention may, if proven successful, be well suited for scaling up.

**Trial registration:**

Current Controlled Trials ISRCTN44599712

## Background

### Implementation of evidence-based knowledge

Evidence-based health care has featured as a policy concern over the last decades. This has been driven by a growing recognition that healthcare practice does not always reflect what is known to be best practice as identified by research evidence. Some studies suggest that thirty to forty per cent of patients do not receive care complying with current scientific evidence [1]. In response to these concerns WHO highlights the necessity of narrowing the research-practice gap and finding ways to ensure that research is translated into clinical practice as effectively and efficiently as possible [2].

Healthcare organisations worldwide face the challenge to increase the utilisation of evidence-based knowledge. The basic assumption is that enhanced use of knowledge, proven to be effective in rigorous research, will improve processes and outcomes in health care. This assumption is supported by a number of studies, maybe most clearly demonstrated in a systematic review of 235 guideline implementation studies where 86% of included studies showed improvements [3]. However, because of the heterogeneity of interventions and strong influence of contextual factors it has not yet been possible to point at what implementation strategies are most effective in what setting. This unpredictability of success of any implementation approach is highlighted in several studies [4,5]. And - most importantly - there is a severe shortage of studies investigating the translation and implementation of knowledge into practice within low- and middle-income countries [6-8]. 'What works where and why' is a global and urgent question for implementation of evidence-based practice.

### Neonatal mortality

Every year almost 4 million newborns die during the neonatal period (the first 4 weeks of life) [9] and another 3 million babies are stillborn, partly related to suboptimal care during pregnancy and childbirth [10]. Action taken towards improved perinatal health and neonatal survival will also contribute to reduced maternal mortality and a reduction in the number of stillbirths. A small set of evidence-based and cost-effective interventions focusing on the mother-child dyad can prevent a major part (up to 72%) of neonatal deaths [11]. Specifically, five interventions targeting the postnatal period have been brought forward: initiation of exclusive breastfeeding, hypothermia prevention and management, kangaroo mother care, pneumonia management and resuscitation [12]. It is imperative to study various strategies to bring these interventions into practice [13].

An important factor for neonatal survival is the recognition of danger signs during pregnancy and the neonatal period, and the timely decision and knowledge to seek care when needed. It is of vital importance for survival chances to reach and receive adequate care in time for mothers and newborns in distress. This is an area in which improvement of knowledge and attitudes is needed at community level. The understanding and mapping of the roles of community actors such as mothers-in-law, husbands, local healers and pharmacies, need to be addressed if delays in reaching health facilities should be shortened [14]. The organisation of the healthcare system, such as commune health centres' provision of care, referral patterns, modes of transportation and costs, also needs to be explored and highlighted in order to improve neonatal health [15]. A combination of community mobilisation and healthcare system approaches is therefore needed.

### Community-based interventions

There is some evidence of effectiveness for community-based interventions to improve perinatal health outcomes in developing countries [16]. Home-based neonatal care in Indian communities has been shown to be effective in reducing neonatal mortality, and a participatory process with women's groups in communities in Bolivia, Guatemala, Indonesia and Nigeria resulted in improvements in referral and reduction in perinatal mortality [17,18]. In an acclaimed article in the *Lancet *in 2004, Manandhar and colleagues reported on a study conducted in Nepal, which, by simple means, succeeded in reducing the risk of neonatal death by 30% (OR 0.70) over a two-year period [19]. The intervention was based on participation in women's groups, where a facilitator stimulated them to identify perinatal health problems that existed in their own environment. By setting up local networks and highlighting issues around childbirth and care of the newborn, a context was created in which information and knowledge could be disseminated in an effective manner, which in turn resulted in positive changes in behavior. The large decline in neonatal mortality that, in this way, was accomplished within a short time indicates that it is possible to make great efforts to improve neonatal health with relatively simple community based interventions [20]. Two other studies, one from India and one from Bangladesh, applied the same method of knowledge translation to improve neonatal health with diverging results. While the intervention was successful in the Indian states of Jhakarand and Orissa [18], it failed to show significant improvement of neonatal mortality rate (NMR) in Bangladesh [21].

### The Vietnamese setting

Vietnam is a developing country which has reported declining levels of maternal mortality [22] and an infant mortality in line with better-off countries [23]. However, similar to many other transitional societies, the level of neonatal mortality has remained largely unchanged in the last three decades, currently constituting nearly three-quarters of all infant deaths [24,25]. A study by our research group found a huge variation of neonatal mortality rates within a province, underlining that high mortality rates and differences in neonatal survival do exist [26]. The Vietnamese Government has identified perinatal health and neonatal mortality as priority areas [27], and evidence-based guidelines on reproductive health (here called the National Guidelines) were launched in 2003, with a revised and exended version presented in 2009 [28]. The presence of these guidelines provided an opportunity to study the uptake of knowledge into practice.

When the present project was initiated in 2005, Vietnam was ranked a low-income country. Due to the rapid economic development in recent years the country was upgraded to a middle-income country in 2010. This transitional stage poses challenges to the health system in terms of accessability for all to the benefits of development [29]. New methods and approaches to meet these challenges and reduce inequities in healthcare provision are needed.

### Theoretical framework

It is increasingly acknowledged that research on knowledge uptake in health care should be theory driven [4,30]. In this proposal we are using the Promoting Action on Research Implementation in Health Services (PARIHS) model as the theoretical framework [31]. This model highlights the importance of three major ingredients for being successful in implementing research into practice: (a) the nature of the evidence being used, (b) the quality of the context in terms of coping with change, and (c) the type of facilitation needed to ensure a successful change process. We are focussing on the third element - facilitation - in this trial, but acknowledging the importance of the two other cornerstones in planning and conducting the study. Facilitation is a function that has been described under many labels, e.g. change agent, opinion leader, research champion, and knowledge broker [32]. Basically, facilitation refers to an approach whereby one person carries out specific tasks and activities to help or make things easier for others [33]. The facilitator role is active and dynamic, concerned with helping, enabling and developing a learning process rather than telling or persuading others about what they should do [34,35]. Stetler and colleagues describes facilitation as a deliberate process of interactive problem solving and support that occurs in the context of recognised needs for improvement and supportive interpersonal relationship [36]. Central in facilitation is to challenge existing practices and support new ways of doing things [37]. Some research is available concerning the effects of facilitators, but findings are not readily interpretable. Based on a review, Harvey et al suggest that a facilitator with face-to-face communication has some impact on changing clinical and organisational practice, although the effect size is variable and associated with differing costs [37]. Thompson et al refers to six intervention studies using external facilitators that were engaged in implementation projects [35]. Findings are equivocal. The facilitation strategy is only marginally tested in the context of developing countries.

### Objectives

The selection of facilitation as the intervention in our study is based on research suggesting that innovations and new knowledge are spread and used as an effect of interaction between individuals and social influence rather than availability of written information [30,35]. We hypothesise that access to evidence-based knowledge, as expressed in the national guidelines, interaction between practitioners and key community members, and a problem-based planned process of change generated through facilitation support, will speed up the process of practitioners changing their behaviour (cluster level). This process will, subsequently, achieve improvements in patient outcomes (individual level). Specifically, we hypothesise that a cluster-randomised intervention using a facilitation approach targeting primary healthcare staff and key community members reduces the risk for neonatal death in the served population, and that the facilitation intervention will result in increased knowledge and use of evidence-based practice related to maternal and perinatal health among healthcare staff in intervention clusters.

### Rationale for study design

The intention with this study is to investigate the implementation of knowledge within and through an existing health system. It is widely recognised that a cluster randomized approach is appropriate for this kind of trial since it is targeted at the health staff rather than at the individual patients [38]. The cluster design is also protective against contamination, both at the facility level as well as at the individual level.

## Methods/Design

### Setting

**Figure 1:**
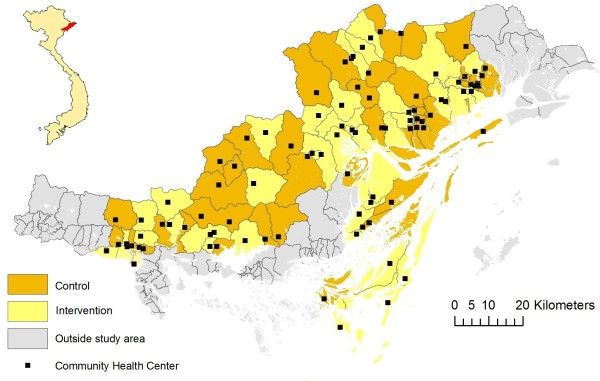
**NeoKIP study area with intervention and control clusters (communes) after randomisation in Quang Ninh province, Vietnam**.

This study was implemented in Quang Ninh province in northern Vietnam, about 120 km east of Hanoi, bordering China in the north (Figure 1). The province inhabits a little more than a million people and is currently undergoing rapid economic development. The ethnic pattern is diverse in Vietnam, with a multitude of ethnic minority groupings, mostly defined by language and culture. In Quang Ninh province, there are ten different ethnic groups (Kinh, Dao, San Chi, San Diu, Tay, Hoa, Cao Lang, Thanh Y, Thanh Phan and Chinee), with Kinh being the majority group constituting about 80% of the population. The province is divided into 14 districts with a wide variety of geographical traits, such as delta land, islands and mountainous areas. Quang Ninh can be considered representative of Vietnam in terms of geography, demography and administrative construction. Basic health care is provided in 13 District Hospitals (DH) and 187 Commune Health Centres (CHC). Antenatal and delivery care is provided at the 187 CHCs, at the 13 DHs and at the two provincial level hospitals.

### Baseline

Based on data covering 2005 [39], there were 17 519 live births occurring in the province, whereof 43% of the deliveries took place at DHs, 16% in CHCs, 32% at provincial hospitals and 8% at home. The neonatal mortality rate (NMR) was 16/1000. The NMR differed substantially between the districts in the province, ranging from 10 to 44/1000 [26]. The 6 districts with the highest mortality had a mean NMR of 28/1000.

### Trial design

The study is a cluster-randomised, community intervention trial evaluating a participatory strategy, conducted by facilitators who collaborate with the local CHC staff and significant community members.

### Participants

**Figure 2:**
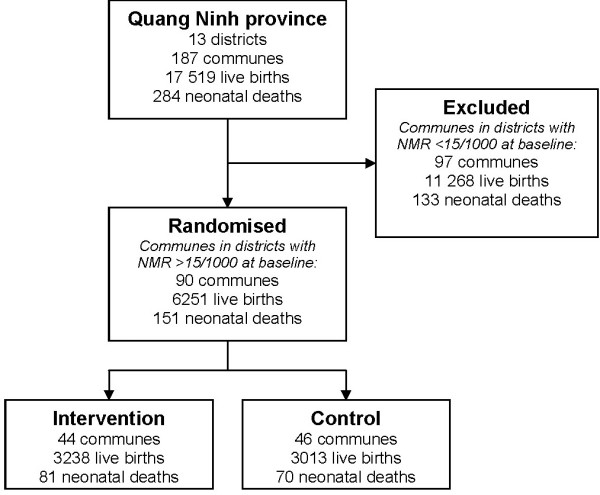
**Participant flow in NeoKIP trial**.

Districts with a NMR higher than 15/1000 were selected to compose the study area, resulting in eight out of 14 districts in the province (Figure 2). In 2005 there were 6251 live births and 151 neonatal deaths in these districts, resulting in a total NMR of 24/1000. These districts were composed of 90 communes with a number of live births ranging from 7 to 270 and neonatal deaths between 0 and 6 (2005). All the 90 communes with a corresponding CHC in the study area were eligible for inclusion, based solely on their geographical location. All newborns delivered within the study area and period were included.

### Intervention

The basic feature of the study intervention is that individuals from the Women's Union (WU) act as facilitators in supporting CHC staff and key community members in their efforts to improve healthcare practices. The WU is an organisation with high national coverage working with various issues related to women's situation. There is a long tradition of involvement at local and regional levels of WU in issues of welfare, particularly in health care matters. A pilot study of the facilitation strategy was performed in 2007. It aimed at practicing training methods of facilitators and test the facilitation approach in real situations. Two individuals from WU were trained and facilitated two group meetings in two communes. The experiences of the pilot told that facilitation was feasible to implement and with a promising potential impact. To initiate the study eight individuals from local WU organisations were recruited and trained for two weeks to be able to act as facilitators. The training program covered topics such as group dynamics, quality improvement methods (e.g. nominal group technique, the Plan-Do-Study-Learn cycle and the Strengths-Weaknesses-Opportunities-Threats diagnostic tool) and basic evidence-based perinatal care. A facilitation manual was developed to guide the work of the facilitators. Two research team members coordinated the facilitation process and acted as supervisor of the facilitators; i.e., supported the facilitators through field supervision and held two-days meetings with all facilitators once a month.

At intervention start a group called the Maternal-Newborn-Health-Group (MNHG) was constituted in each commune. This group consisted of three CHC staff, a village health worker, a population motivator, the vice chairman in the commune and WU representatives from a village and from the community level. The facilitators' mission during the intervention was to support MNHG members to identify problems and empower them in improving perinatal practice. Each facilitator operated within the same communes the whole intervention period and met each MNHG monthly. Basing the MNHG discussions on individual and common experiences in the local setting, the facilitator supported the group in critical reflection, problem identification, finding solutions, and developing change strategies. This intervention strived to achieve a local ownership and 'bottom-up' approach in empowering healthcare staff to improve practice. When appropriate, the facilitators would try to highlight recommendations in the National Guidelines [28]. The intervention took place at cluster level, aiming to achieve improvements in neonatal health and survival at the individual level.

### Data collection and outcome

Data were collected both on cluster level as well as on individual level on three main domains; the intervention process (cluster level), perinatal healthcare process indicators (cluster and individual level), and the primary outcome of the intervention, being neonatal mortality (individual level) (Table 1).

**Table 1:** Outcomes and methods of data collection for evaluation of the NeoKIP project in Quang Ninh province, Vietnam

	Variables	Methods of data collection
**Intervention process**	• *Intervention coverage*; number of participants at intervention meetings, topics discussed at meetings, frequency of meetings • *Intervention process*; number of identified problems relating to perinatal health, progress in working with these problems, interaction between group and facilitator, methods used during process	• Focus groups discussion with facilitators and healthcare staff • Meeting protocols • Facilitators' diaries • Individual interviews with MNHG participants and healthcare staff

**Healthcare process data**	• *Antenatal care usage*; frequency and timing of antenatal care, ultrasound examiniation rate, antenatal care qualiy measurement. • *Delivery care utilisation*; delivery prepardeness, home delivery rate and care-seeking patterns. • *Delivery care*; caeserean section rate, transfer patterns, assistance at delivery. • *Immediate postnatal care at place of delivery *; resuscitation rate, temperature control, breast-feeding initiation, rate of exclusive breast-feeding at two months. • *Postnatal care at home*; umbilical care, prevalence and duration of skin-to-skin, exclusive breast-feeding rate and frequency and timing of home visits by midwife. • *Causes of neontal death* • *Healthcare resources*; healthcare staff knowledge on perinatal care, availability of equipment and drugs at health facilities • *Sex ratio at birth*	• Surveillance • Audits every six months at health facilities • Case-referent interviews • GIS • Knowledge assessment survey

**Primary outcome**	• *Neonatal mortality*	• Surveillance • Case-referent interviews • GIS

#### The intervention process

The intervention process was monitored continuously. Issues like MNHG's choice of improvement topics, activities for improving practice, the interaction between facilitators and group members, and progress of the facilitation process at all intervention sites were examined using several approaches, like interviews with facilitators and focus group discussion with MNHG members, analyses of facilitator's diaries from MNHG meetings and the notes from the monthly meetings with the supervisor.

#### Healthcare process

Data on reproductive and perinatal health indicators were collected through routine registration within the study districts by data collectors employed by the project. They attended monthly meetings at the CHCs where village health workers report to CHC staff and visited district hospitals and the provincial hospital in Ha Long city and UBGH once a month to collect information on births and deaths from the study area. They also conducted an audit of equipment and drugs at all health facilities every six months.

When a case of neonatal death was ascertained, a home-based interview with the mother who had lost a child (estimated at 150/year) was conducted. Through a randomisation process 6% of the total population of live births were selected as referents and their mothers were interviewed. Interviewers employed by the project conducted these interviews using a semi-structured interview form, collecting information on family characteristics and perinatal narratives. A comparison, based on interviews and routine data collection, between intervention and control communes will be performed for perinatal process indicators. During the first two years of intervention, July 2008 until June 2009, 233 out of 238 mothers who had lost a newborn in the neonatal period were interviewed. During the same time period 813 referent mothers, representing 5.6% of the total 14 453 live births within the study area, were interviewed.

Geographic location of all health facilities and homes of neonatal deaths and referents were collected by setting up a Geographic Information System (GIS). Spatial analyses, including cluster analysis and analysis of distances will be applied when appropriate.

A questionnaire for assessing staff knowledge was developed by the research team that consists of 16 multiple-choice questions covering five basic areas of evidence-based practice in neonatal care: breastfeeding, immediate postnatal care, infection management, low birth weight management and postnatal home visits. The choice of topics was based on the national guidelines [28] and WHO recommendations on newborn health care [40]. An extended and modified version of the questionnaire have been used before the intervention and was used after the intervention in both intervention and control clusters to see if the intervention has had any effect on staff knowledge. The late knowledge assessment will be accompanied with case vignettes to assess how staff will act in different scenarios.

#### Primary outcome

The primary outcome, neonatal mortality, will be evaluated using the surveillance data and case-referent interviews described above. GIS-based analyses of neonatal mortality will also be performed.

### Sample size

Power calculations for sample size were based on neonatal mortality being a primary outcome measure. The design effect was assumed to be 1.5 for the purpose of sample size calculation. Given the level of current neonatal mortality of 24/1000 in 2005 the study size has sufficient power to demonstrate a significant reduction of 7/1000 and more over the three year study period, i.e. to 17/1000 or less (alfa 0.05, beta 0.2). Using a case-referent approach for the evaluation a three-year sample of neonatal deaths (450 cases) and randomly selected referents, 6% from the total population of live births (1120 referents), will allow to demonstrate an effect of the randomised intervention of 26% (OR 0.74, 95% CI 0.55-0.99).

### Randomisation

The cluster of intervention, and thus the unit of randomisation, was the commune and its corresponding health station. Probability proportional to size sampling (PPS) based on the total number of live births at baseline (2005) was used. Randomisation continued until one arm had reached over 50% of the total amount of live births. This resulted in 44 out of 90 clusters being allocated to intervention (Figure 2). Randomisation was performed by the research team.

### Data analysis

#### Intervention process

The intervention process will be analysed at cluster level. Qualitative analyses of the intervention process, through methods described above, are to be performed through content analysis. Quantitative data, like meeting attendance and frequency and measurements of knowledge level among health staff will be analysed to estimate the effect of the intervention.

#### Healthcare process and primary outcome

The healthcare process will be analysed at individual level, using the case-referent design previously described. By applying a case-referent design it will be possible to adjust for possible confounding. The primary outcome, neonatal mortality, which also will be analysed at individual level, comparing the intervention and control area using surveillance data as well as case-referent data. These analyses will use statistical techniques to account for the cluster design as suggested by Campbell et al, with an appropriate adjustment for design effect [38].

### Time plan

The project, which has been given the acronym NeoKIP (Neonatal health - Knowledge Into Practice) started in autumn 2005 by negotiations with provincial health authorities and representatives of the WU in Quang Ninh. Baseline data collection, covering year 2005 data, was performed in 2006. The facilitation intervention was initiated in July 2008 and continued until June 2011. Analysis of the intervention process is ongoing and analyses of outcome data will take place in 2011-12.

### Dissemination of project results

Previously published results from the NeoKIP project has reveled a substantial under-reporting of neonatal deaths, with a NMR at baseline being four times higher than what was officially reported [41]. Baseline investigations also found a strong correlation between home deliveries and neonatal death [39] and that 25% of the newborns that died during 2005 did not have any contact with the health system prior to death [42]. In the baseline study we used the knowledge assessment questionnaire mentioned above in a survey, with results indicating a clear potential for improvement in basic neonatal knowledge at primary healthcare level [43]. Focus group discussions with primary healthcare staff during the baseline study indicated that primary healthcare staff work in a context that only to some extent enables them to translate knowledge into practice [44].

Furthermore, analyses of data collected during the intervention period has demonstrated that ethnic minority mothers are at increased risk of experiencing a neonatal death [45] and that there is marked distance decay in delivery care utilisation in this setting [46], indicating target areas for the intervention.

Future results from the trial will be published in peer-reviewed international journals and presented at international conferences. The findings and its implications has been and will continue to be reported to and disseminated through the Ministry of Health in Vietnam. Results will be taken into consideration for future policy and planning. The approach addressing this kind of intervention to an existing healthcare system has not been scientifically studied before in a resource poor setting and the results will be of great interest for the public health community. Finding ways to make progress can be anticipated to have a global audience.

### Ethical considerations

Participating in the study can involve stressful moments for participating families as families having neonatal deaths will be visited for an interview. Informed consent will be asked from all families before interviews and all staff within the project will work under confidentiality to protect respondents. Anonymity of subjects was secured through depersonalisation of data at an early stage of data handling. The study was approved by the Ministry of Health in Vietnam, the Provincial Health Bureau in Quang Ninh and the Research Ethics Committee at Uppsala University, Sweden (Dnr 2005:319).

## Discussion

To find effective and feasible methods of knowledge translation in order to improve maternal and newborn health and survival is a global research priority and essential for reaching the Millennium Development Goals 4 and 5. A lot of attention has been given to community participation as a driving force of change, and trials with women's groups have shown encouraging results in relation to neonatal survival [18,19]. There is however an equally great need to find ways to scale up interventions of knowledge uptake that have been proven effective in a relatively small sample considering the magnitude of the problem. The successful trials in Nepal and India have been performed with a high coverage and intense activities in the intervention clusters. In order for this approach to be scaled up it will need to be carried out in existing health systems. Therefore, our study intends to evaluate a method of knowledge translation which is less resource and time demanding and that can be applicable within incumbent healthcare structures.

Previous studies using community-based participatory approaches in order to improve neontal survival have been situated in settings with a rather high NMR. The panorama of causes of neonatal death and the subsequent means for averting them differs between different strata. In high-mortality settings there will be a larger proportion of deaths due to infections, and community-based interventions like the recognition of danger signs and improved care seeking patterns is a priority, whereas health system strengthening interventions like access to emergency obstetric care (EmOC) will be needed in order to lower NMR further in settings in the lower NMR strata. Our study is set in a setting with a NMR well below the global average, but still in an interval where community-based interventions is expected to be needed for improving neonatal survival. As such, the present study fills a scientific gap not only in the Vietnamese setting but is of global interest.

The intervention approach - team based facilitation - was informed by the PARIHS framework and adapted to the Vietnamese context. At a first instance it might look odd to apply a "bottom-up" approach in a context like the Vietnamese which is characterised of "top-down" gouvernance. However, testing this approach was met by enthusiasm in the project team and other Vietnamese stakeholders. A pilot study further indicated that facilitation was feasible and promising as an intervention. The concrete shape of the intervention was developed and performed by the Vietnamese collaborators. Issues like who to include in the groups that would receive facilitation and who that would be a suitable facilitator were carefully considered, having the opportunities to upscaling in mind. Thus, in the evaluation stage of the study we put particlular emphasis on understanding how the facilitation intervention is working in this specific Vientamese context. A great deal of the data collection is about shedding light on how the facilitators perform their role and how they are perceived by involved stakeholders. While we explicitly have used the PARIHS framework to design the study we will be able to provide feedback on how this model works, particularly the facilitation cornerstone, in a cultural context different from where the framework was developed.

Additional to this professional approach, the current project has a profound gender perspective. It recognises the local gender power structure within which women live and which may circumvent their ability to improve health practices. It acknowledges the vital role of mothers as actors in improving their own and their newborns' health. The majority of the healthcare workers responsible for perinatal care at primary level are women. By employing female facilitators (women recruited from the local WU) to empower local health workers and the mothers, the project takes advantage of a shared female knowledge and agency across the main actors involved in the intervention. We believe this combined professional and female actor's focus increases the potential for successful outcomes and sustainable effects.

The findings will not only be of interest in Vietnam and other countries with a similar healthcare context, but also in high-income settings. Rapidly increasing costs for health care, enhanced demands from the patients, and an exponentially growing knowledge base for health care makes it necessary to develop evidence-based interventions for knowledge uptake. The global relevance of effective knowledge translation strategies and the potential for substantial improvements of people's health make it urgent to accomplish well designed studies in this field.

## Competing interests

The authors declare that they have no competing interests.

## Authors' contributions

LÅP, NTN, DPH, MM, AJ and UE conceptualised and designed the study. LW and LE provided input on the design. UE, LÅP and NTN were principal applicants for funding. LW, LE and TQH designed the intervention. LW and MM drafted the study protocol manuscript. All authors provided intellectual input and and approved the final manuscript.

## Pre-publication history

The pre-publication history for this paper can be accessed here:

http://www.biomedcentral.com/1472-6963/11/239/prepub
